# Ruptured Hepatic Carcinoma After Transcatheter Arterial Chemoembolization^☆^

**DOI:** 10.1016/j.curtheres.2012.12.006

**Published:** 2013-06

**Authors:** Zhongzhi Jia, Feng Tian, Guomin Jiang

**Affiliations:** Department of Interventional Radiography, Second Hospital of Changzhou Affiliated With Nanjing Medical University, Changzhou, Jiangsu Province, China

**Keywords:** transcatheter arterial chemoembolization, hepatic carcinoma, retreatment, rupture

## Abstract

**Background:**

Transcatheter arterial chemoembolization (TACE) is recommended as the first-line therapy for unresectable hepatic carcinoma (HCC). Serious complications such as hepatic abscess and hepatic decompensation are well known, but rupture of HCC after TACE is a rare complication.

**Objective:**

The aim of this study was to identify the associated risk factors and the outcomes resulting from ruptured HCC after TACE.

**Methods:**

A retrospective analysis was performed in 6 patients who experienced ruptured HCC after TACE.

**Results:**

All patients underwent chemoembolization after superselective catheterization of the appropriate hepatic artery. The interval between the treatment of TACE and ruptured HCC was 6 to 17 days (mean [SD] 10.33 [4.08] days). Common features in ruptured HCC were large tumor size, location of the tumor adjacent to liver capsular membrane, and complete occlusion of the tumor feeding artery, especially in those with a large amount of iodized oil plus polyvinyl alcohol particles. Two patients underwent emergency embolization, and 4 patients received conservative treatment. Except for 2 patients treated conservatively who died 45 and 68 days after ruptured HCC with hepatic decompensation, the other 4 patients survived to the 6-month follow-up.

**Conclusions:**

Ruptured HCC after TACE is a rare but serious complication. Large tumor size, location of the tumor adjacent to the liver capsule, and complete occlusion of the feeding artery may be predisposing factors. More research is needed to examine which patients presenting with ruptured HCC after TACE would benefit from conservative or emergency arterial embolization procedures.

## Introduction

Hepatic carcinoma (HCC) is one of the most common malignant tumors and the third leading cause of cancer mortality in the world.^1^ Transarterial chemoembolization (TACE) is one of the most common treatment modalities, being a palliative and preoperative method for patients with HCC who are considered to be unsuitable candidates for surgery.^2^ Although satisfactory survival results can be achieved with TACE, it is associated with complications causing significant morbidity and mortality. Numerous publications describe minor complications associated with TACE in 10% to 12% of patients.^3–8^ These include postembolization syndrome (fever, abdominal pain, nausea, and vomiting), impaired liver function, and leukocytopenia.^3,6^ Although these are common, there are very few reports of severe post-TACE complications in the literature.^6–8^ Major complications include a 3% rate of irreversible liver failure and liver abscess, upper gastrointestinal bleeding, bile duct complications, acalculous cholecystitis, pulmonary embolism, spasm or occlusion of the hepatic artery, and acute renal failure. Ruptured HCC after TACE has rarely been reported. Here, we report our experience with ruptured HCC post-TACE in 6 patients.

### Patients

Six consecutive patients (all men; 45–67 years of age, mean [SD] age, 57.83 [8.01] years) fulfilled diagnostic criteria for ruptured HCC after TACE, as demonstrated by Doppler ultrasound and computed tomography scan or paracentesis, were referred to our center from May 2008 to May 2011. The mean (SD) tumor size was 13.17 (2.79) cm (range, 9.0–16.0 cm). Two patients (33.3%) had Child-Pugh grade A hepatic impairment, and the remaining 4 patients (66.7%) had hepatitis B grade impairment. All patients provided signed informed consent.

## Methods

A retrospective analysis was performed of the patients who underwent chemoembolization from May 2000 to December 2012. All patients were regularly followed at outpatient clinics. alpha-fetoprotein, Doppler ultrasound, or computed tomography scan was performed 1, 3, and 6 months post-TACE. The clinical parameters and radiological features of the tumor in patients with rupture were reviewed.

## Results

All patients underwent chemoembolization with a calculated dose of oxaliplatin (100–150 mg) and hydroxycamptothecin (8–10 mg) based on body weight and liver function after superselective catheterization of the appropriate hepatic artery. A mixture of pirarubicin and iodized oil⁎ (10 mg pirarubicin and 10 mL iodized oil) was injected into the arterial branches until hemostasis was achieved (18–30 mL; mean, 23.3 mL). Gelatin sponge particles were used in patients whose tumor had a rich blood supply.

In our study, 6 patients had ruptured HCC after TACE, and the interval between the TACE treatment and ruptured HCC was 6 to 17 days (mean [SD], 10.33 [4.08] days). The symptoms and signs of rupture include sudden onset of severe abdominal pain in all patients and the presence of blood in the peritoneal cavity in the endemic area in 4 patients. None had radiological evidence of arteriovenous fistula, coagulopathy, sepsis, or toxicity from TACE. Two patients had emergency embolization, and the remaining 4 patients received conservative treatment (medications for pain relief, hemostasis and energy input, and rest). Except for 2 patients treated conservatively who died 45 and 68 days after ruptured HCC with hepatic decompensation, the other 4 patients survived to a 6-month follow-up in stable condition. The clinical parameters and treatment outcomes are summarized in Table I.

## Discussion

Rupture of HCC after TACE is a life-threatening condition that may cause severe bleeding and hypotension, especially in cirrhotic patients who have coagulation deficiencies. Therefore, the mortality rate of this catastrophic event is very high. It is important to identify ways to reduce the mortality associated with ruptured HCC after TACE.

The mechanism of rupture of HCC after TACE is not fully understood. It is presumably due to tumor and capsular necrosis, increased pressure inside the tumor, vascular injury during TACE, or inflammation secondary to the chemotherapeutic agents.^9^ In addition, several factors such as direct trauma, high pressure due to edema of the tumor, erosion of a vessel, occlusion of the hepatic veins by tumor thrombus, and coagulopathy have been accepted as causes.^10^ A peripherally located tumor has been observed in several studies to rupture more easily than one surrounded by normal parenchyma.^11,12^ The size and degree of extrahepatic protrusion have also been found to be predictive of rupture risk.^11–13^ It had been reported that large tumor size or extracapsular extension of the tumor appeared to be a predisposing risk factor.^9,11,12^ Because typically HCC is a hypervascular tumor that contains many abnormal blood vessels, any small increase in vascular load owing to portal hypertension or mechanical injury may result in tears in the vessel wall producing a hematoma. Moreover, the arterial wall is probably not normal in patients with portal hypertension because the incidence of visceral artery aneurysms is 2% to 4% in patients with cirrhosis.^9^ Tumor necrosis caused by TACE may be exaggerated by a secondary infection. Necrosis and the presence of hematoma will result in increased intratumoral pressure causing rupture.^9^

Ruptured HCC after TACE should be suspected in patients presenting with sudden onset of severe abdominal pain, abdominal distention, shock, and the presence of blood in the peritoneal cavity in an endemic area.^14^ Diagnosis could be confirmed by paracentesis and angiography. As seen in our study, all 6 patients presented with sudden onset of severe abdominal pain, and in 4 patients, blood was present in the peritoneal cavity in an endemic area.

The common features in the 6 patients with ruptured HCC in our study were large tumor size, location of tumors adjacent to the liver capsule, and the use of a large amount of iodized oil plus polyvinyl alcohol particles to completely embolize the artery feeding the tumor. These features may be predisposing factors in ruptured HCC after TACE.

The primary objective in the management of these patients is to achieve hemostasis by surgery, embolization, or conservative methods. The mortality and morbidity rates are high among patients with ruptured HCC because these patients usually have a poor liver reserve and advanced disease. In our experience, repeat embolization can be performed to stabilize the patient’s condition and the tumor in patients whose blood pressure is unstable, and conservative treatment can be used for patients whose blood pressure is stable after ruptured HCC after TACE to get long-term relief.

## Conclusions

Ruptured HCC after TACE is a rare but potentially life-threatening complication. Large tumor size, location of tumors adjacent to the liver capsule, and complete occlusion of the tumor feeding artery, especially in patients with a large amount of iodized oil plus polyvinyl alcohol particles may be predisposing factors for HCC rupture after TACE. Further prospective research is required to fully understand its mechanism.

## Conflicts of Interest

The authors have indicated that they have no conflicts of interest regarding the content of this article.

## Figures and Tables

**Table I:** Clinical parameters and treatment outcome.

Variable	Patient					
	1	2	3	4	5	6
Age, y	45	61	53	57	64	67
HBsAg	+	−	+	+	−	−
Cirrhosis	Yes	No	Yes	No	No	No
Tumor size, cm	9	13	11	14	16	16
Location (lobe)	Right	Right	Right + left	Right + left	Right + left	Right + left
Near liver capsule	Yes	Yes	Yes	Yes	Yes	Yes
Tumor capsule	No	Yes	No	Yes	Yes	Yes
Embolism material	L+G	L+G+P	L+P	L+P	L+P	L+G
Amount of iodized oil, mL	20	18	20	25	27	30
PVTT	No	No	Yes	Yes	No	No
Interval between TACE and rupture, days	10	6	7	9	17	13
Outcome	Died	Alive	Died	Alive	Alive	Alive

G, gelatin sponge particles; HBsAg, surface antigen of hepatitis B; L, iodized oil; P, polyvinyl alcohol particles; PVTT, portal vein tumor thrombi; TACE, transcatheter arterial chemoembolization.

## Footnotes

* Trademark: Lipiodol^®^ (Guerbet, Cedex, France).
